# Role of Erythromycin-Regulated Histone Deacetylase-2 in Benign Tracheal Stenosis

**DOI:** 10.1155/2020/4213807

**Published:** 2020-01-17

**Authors:** Zhenjie Huang, Peng Wei, Luoman Gan, Tonghua Zeng, Caicheng Qin, Guangnan Liu

**Affiliations:** ^1^Guangxi Medical University, Nanning, China; ^2^Department of Respiratory Medicine, The Second Affiliated Hospital of Guangxi Medical University, Nanning, China; ^3^Medical School of Qinghai University, Xining, China; ^4^Department of Respiratory Medicine, Beihai People's Hospital, Beihai, China

## Abstract

**Objective:**

This study aims to explore the role of erythromycin-regulated histone deacetylase-2 in benign tracheal stenosis.

**Methods:**

The rabbit model of tracheal stenosis was established. The rabbits were randomly divided into 8 groups. Histone deacetylase-2 (HDAC2) expression was detected by immunofluorescence. The expression of type I collagen and type III collagen was detected by immunohistochemical method. The expression of TGF-*β*1, VEGF and IL-8 in serum and alveolar lavage fluid was detected by ELISA. The expression of HDAC2, TGF-*β*1, VEGF and IL-8 in serum and alveolar lavage fluid was detected by ELISA. The expression of HDAC2, TGF-

**Results:**

In Erythromycin (ERY) group, ERY + Budesonide group, ERY + Vorinostat group and ERY + Budesonide + Vorinostat group, the degree of bronchial stenosis was alleviated, and the mucosal epithelium was still slightly proliferated. The effect of ERY combined with other drugs was more obvious. The HDAC2 protein expression increased significantly in ERY group, ERY + Budesonide group and ERY + Budesonide + Vorinostat group and decreased significantly in Vorinostat group, the expression of collagen I and III decreased significantly in ERY group, ERY + Budesonide group and ERY + Budesonide + Vorinostat group (*P* < 0.05). The TGF-*β*1, VEGF and IL-8 in serum and alveolar lavage fluid was detected by ELISA. The expression of HDAC2, TGF-*P* < 0.05). The TGF-

**Conclusions:**

Erythromycin inhibited inflammation and excessive proliferation of granulation tissue after tracheobronchial mucosal injury by up-regulating the expression of HDAC2, it promoted wound healing and alleviated tracheobronchial stenosis. When combined with budesonide, penicillin and other glucocorticoids and antibiotics, it had a good synergistic effect. However, vorinostat could attenuate erythromycin's effect by down-regulating the expression of HDAC2. It may have good clinical application prospects in the treatment of tracheal stenosis.

## 1. Introduction

Benign airway stenosis refers to a common clinical disease in which tracheal and bronchial stenosis, or even complete obstruction, results from excessive proliferation of granulation tissue in the process of repeated self-repair after long-term stimulation and injury of tracheal mucosa. Its pathological mechanism is mainly caused by injury of tracheal mucosa and inflammation during trauma, operation or intubation. Fibroblasts begin to proliferate and migrate under the stimulation of inflammatory mediators and growth factors, increase the production of extracellular matrix, and finally make granulation tissue form scar tissue proliferation [1, 2].

Tracheal balloon dilatation is a safe, effective and economical method for the treatment of benign tracheal stenosis, the effective rate is as high as 100%, but the restenosis rate after treatment is as high as 40–70% [3]. These patients often need repeated endoscopic interventional therapy, which significantly increases the patient's pain, treatment risk and financial burden, which has become a major problem facing clinicians.

At present, the reported methods for preventing and treating restenosis after endoscopic interventional therapy for tracheobronchial stenosis include local application of mitomycin C, intraluminal brachytherapy or drug-coated stent, cryotherapy and other cold interventional methods, full anti-infective therapy [4–8]. Histone deacetylase-2 (HDAC2) can inhibit the activity of nuclear factor-*κ*B (NF-*κ*B) and then inhibit the expression of inflammatory factors [9]. Low-dose erythromycin can play an anti-inflammatory role by up-regulating the activity of HDAC2 [10].

The anti-inflammatory erythromycin, budesonide and penicillin are drugs for positive regulation of HDAC2, while Vorinostat (HDAC2 inhibitor) is a drug for negative regulation of HDAC2 [11]. The interference of HDAC2 inhibitor can block the effect of erythromycin and other anti-inflammatory drugs on tracheal inflammation and affect the occurrence of tracheal stenosis after tracheal injury.

In this study, HDAC2 inhibitor combined with different anti-inflammatory drugs to study the effects of erythromycin or erythromycin combined with other anti-inflammatory drugs on tracheal stenosis, the changes of inflammatory factors and cytokines in experimental animals and their relationship, to further understand the effect of HDAC2 on airway inflammation and its regulation mechanism, which provide theoretical basis and new ideas for clinical prevention and treatment of tracheal stenosis after injury.

## 2. Materials and Methods

### 2.1. Experimental Animals

A total of 48 New Zealand rabbits (4 weeks old) were purchased from Nanchang Longping Rabbit Industry Co., Ltd. They were bred in the SPF class barrier system. They were maintained in a temperature controlled room (18–22°C) with 12-hr light/dark cycles, eat and drink freely.

### 2.2. Experimental Reagents and Instruments

Erythromycin enteric-coated tablets (H42021990, Yichang Humanwell Pharmaceutical Co., LTD.); Vorinostat Capsules (180509, Beijing Hengrui Kangda Medical Science and Technology Development Co., Ltd.); Budesonide (AstraZeneca 8339000); Rabbit Anti-Collagen III Polyclonal Antibody (bs-10423R, Bioss); Rabbit Anti-Collagen I Polyclonal Antibody (bs-0549R, Bioss); Rabbit Anti-HDAC2 Polyclonal Antibody (bs-1813R, Bioss); Rabbit VEGF ELISA kit (MM-021001); Rabbit TGF-*β*1 ELISA kit (MM-3684001); Rabbit Polyclonal Anti-VEGF (bs-1313R, Bioss, 1/500–1/2000); Rabbit Polyclonal Anti-TGF*β*1 (bs-0086R, Bioss, 1/500–1/2000); Rabbit monoclonal Anti-IL-8 (ab34100, abcam, 1/1000); Rabbit Polyclonal Anti-HDAC2 (OmnimAbs, OM105905, 1/500–1/2000); fluorescence microscope (CKX53, OLYMPUS); Microplate Reader (RT-6100, Rayto); Protein vertical electrophoresis instrument (DYY-6C, Beijing 61 instrument factory); Ultra High Sensitivity Chemiluminescence Imaging System (Chemi DocTM XRS+, Bio-Rad Shanhhai Laboratories).

### 2.3. Establishment of Tracheal Stenosis Model

The rabbits had to fast for 8 hours before modeling. Each rabbit was anesthetized with an intravenous injection of 3% pentobarbital sodium (1 ml/kg) and placed supine on an operating table. To enhance analgesia, 2% lidocaine hydrochloride was injected into the anterior neck. After anesthesia, the rabbits were supine and fixed on the operating table. Skin preparation in anterior cervical region, it was disinfected with 0.5% iodophor twice. Longitudinal incision of skin was about 4-5 cm, subcutaneous tissue and muscle was separated layer by layer to expose trachea, annular tracheotomy was performed in cartilage space 3 and 4, the length was 2/3 of the circumference of trachea and avoided injury of tracheal cartilage. The proximal end of trachea was lifted to avoid suffocation caused by blood flow back to the distal end of trachea, bleeding was stop by compressing tracheal incision. The rigid nylon brush was inserted into the distal trachea about 1.5 cm through the incision, rubbed back and forth 20 times on the front and side walls of the trachea. If intratracheal hemorrhage occured during friction, gauze was used to compress hemostasis. After no obvious hemorrhage, No. 4.0 single thread was used to suture the trachea intermittently for 3 needles, muscle layer, subcutaneous tissue layer and skin layer by layer carefully. Disinfect the wound again and covered the wound with sterile gauze. They were taken back to the cage when they were awake naturally.

### 2.4. Experimental Grouping

They were divided into 8 groups. There was no treatment in Control group. In Normal saline (NS) group, they were treated with penicillin (4.35 million U/kg) twice daily by intramuscular injection and normal saline (15 ml) twice daily by aerosol inhalation. In Erythromycin (ERY) group, they were treated with penicillin (4.35 million U/kg) twice daily by intramuscular injection and Erythromycin (13.6 mg/kg) twice a day by gavage administration. In Budesonide group, they were treated with penicillin (4.35 million U/kg) twice daily by intramuscular injection and budesonide suspension (0.05 mg/kg) twice a day by atomization inhalation. In ERY + Budesonide group, they were treated with penicillin (4.35 million U/kg) twice daily by intramuscular injection, Erythromycin (13.6 mg/kg) twice a day by gavage administration and budesonide suspension (0.05 mg/kg) twice a day by atomization inhalation. It is reported that the doses of Vorinostat used in animal experiments are generally between 30 mg/kg and 50 mg/kg [12]. We carried out different doses of Vorinostat and found that 40 mg/kg dose had an ideal inhibition effect on HDAC2. In Vorinostat group, they were treated with penicillin (4.35 million U/kg) twice daily by intramuscular injection and Vorinostat capsule 40 mg/kg once a day. In ERY + Vorinostat group, they were treated with penicillin (4.35 million U/kg) twice daily by intramuscular injection, Erythromycin (13.6 mg/kg) twice a day by gavage administration and Vorinostat capsule 40 mg/kg once a day. In ERY + Budesonide + Vorinostat group, they were treated with penicillin (4.35 million U/kg) twice daily by intramuscular injection, budesonide suspension (0.05 mg/kg) twice a day by atomization inhalation and Vorinostat capsule 40 mg/kg once a day. There were six rabbits in each group, samples were taken and tested after 10 consecutive days of administration.

### 2.5. HE Staining Test

The tissues were taken and washed with PBS, then they were fixed with 4% paraformaldehyde solution and embedded in paraffin. They were cut into 5 *μ*m slices and stained with HE using conventional method. Briefly, Deparaffinize sections, 2 changes of xylene, 10 minutes each; Re-hydrate in 2 changes of absolute alcohol, 5 minutes each; 95% alcohol for 2 minutes and 70% alcohol for 2 minutes; Wash briefly in distilled water; Stain in Harris hematoxylin solution for 8 minutes; Wash in running tap water for 5 minutes; Differentiate in 1% acid alcohol for 30 seconds; Wash running tap water for 1 minute; Bluing in 0.2% ammonia water or saturated lithium carbonate solution for 30 seconds to 1 minute; Wash in running tap water for 5 minutes; Rinse in 95% alcohol, 10 dips; Counterstain in eosin-phloxine solution for 30 seconds to 1 minute; Dehydrate through 95% alcohol, 2 changes of absolute alcohol, 5 minutes each; Clear in 2 changes of xylene, 5 minutes each; Mount with xylene based mounting medium. They were observed under optical microscope.

### 2.6. Immunohistochemical Detection

Briefly, the tissues were embedded with paraffin using conventional method. They were cut into 5 *μ*m slices, and incubated with 0.3% endogenous peroxidase blocking solution for 20 min after dewaxing and hydrating. Then they were incubated at room temperature for 10 min with 3% hydrogen peroxide methanol solution, and washed with PBS for 3 times (3 min/time). Antigen retrieval was performed using citrate buffer (pH 6.0) at 121°C for 2 min. After blocking with 5% BSA (Gibco; Thermo Fisher Scientific, Inc.), they were incubated with a primary monoclonal antibody overnight at 4°C. They were then incubated with goat anti-rabbit non-biotinylated regents (Zhongshanjinqiao, Beijing, China) according to the manual and mounted with epoxy resin.

### 2.7. Immunofluorescence Assay

The tissue slices were baked in an oven at 65°C for 2 hours. They were put into dimethylbenzene for 10 min, and then they were put into 100% ethanol, 100% ethanol, 95% ethanol, 80% ethanol and purified water in turn for 5 minutes each. Antigen retrieval was performed using citrate buffer (pH 6.0) at 121 C for 2 min. After blocking with 5% BSA (Gibco; Thermo Fisher Scientific, Inc.), they were incubated with a primary monoclonal antibody (1  :  400 HDAC2) overnight at 4 C. The slices were washed with PBS for three times and incubated with fluorescent antibody Cy3 (1  :  200) at 37 C for 30 min. DAPI was added into them and incubated for 5 min avoiding light. Excess DAPI was washed with PBS and then washed with water for 1 min. The slices were mounted with mounting medium with anti-fluorescence quenching agent.

### 2.8. ELISA Assay

The levels of VEGF, IL-8 and TGF-*β*1 in different groups were detected using ELISA kits according to the manufacturer's instructions. Optical density values at 450 nm were determined by a Microplate Reader (RT-6100, Rayto).

### 2.9. Western Blotting Detection

Total proteins were extracted and protein concentration was determined using BCA. Proteins (50 *μ*g per lane) were separated using 12% SDS-PAGE, then they were electrotransferred to a PVDF membrane (Amersham Biosciences, Piscataway, NJ, USA). The PVDF membrane was rinsed with TBS for 10–15 min, placed in TBS/T blocking buffer containing 5% (w/v) skimmed milk powder. It was incubated at room temperature for 2 h following the addition of an appropriate dilution of primary antibodies. The membrane was then rinsed with TBST three times (5–10 min/wash) and then incubated at room temperature for 1 h with horseradish peroxidase-labeled secondary antibody (1  :  50,000; Abcam, Cambridge, UK; diluted with TBST containing 0.05% (w/v) skimmed milk powder). The membrane was then rinsed three times with TBST (5–10 min/wash). Protein bands were detected using an enhanced chemiluminescence kit (Perkin-Elmer Inc.) and quantified as the ratio to GAPDH. Quantification was performed using “Quantity one” software.

### 2.10. Statistical Analysis

The data were analyzed using SPSS 19.0 software (SPSS Inc., Chicago, IL, USA). All results are presented as the mean ± standard deviation (SD) and one-way ANOVA or Student's *t*-test were used for comparison between groups. *P* < 0.05 was considered to indicate a statistically significant difference.

## 3. Results

### 3.1. The Model Was Successfully Established

**Figure 1:**
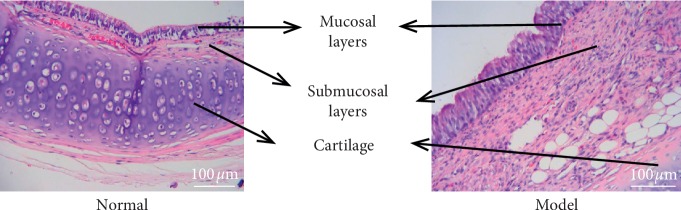
Pathological results. There was no hyperplasia of trachea tissue in Normal group, while bronchial cavity stenosis, tissue hyperplasia and mucosal epithelial hyperplasia were observed in the model group.

As shown in Figure 1, no hyperplasia of trachea tissue was found in Normal group. In the model group, bronchial cavity stenosis, tissue hyperplasia and mucosal epithelial hyperplasia were observed. It showed that the model was successfully established.

### 3.2. HE Staining Results

**Figure 2:**
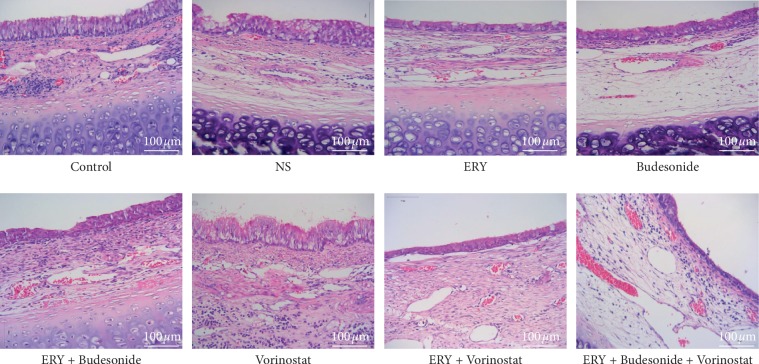
HE staining results. Control: rabbit tracheal stenosis model without treatment; NS: rabbit tracheal stenosis model treated with penicillin; ERY: rabbit tracheal stenosis model treated with erythromycin; Budesonide: rabbit tracheal stenosis model treated with budesonide; Vorinostat: rabbit tracheal stenosis model treated with vorinostat.

As shown in Figure 2, in control group, NS group, Budesonide group and Vorinostat group, bronchial cavity stenosis, tissue hyperplasia and mucosal epithelial hyperplasia were observed. In ERY group, ERY  + Budesonide group, ERY  +  Vorinostat group and ERY  + Budesonide  +  Vorinostat group, the degree of bronchial stenosis was alleviated, and the mucosal epithelium was still slightly proliferated. The effect of erythromycin combined with budesonide was more obvious than that of others.

### 3.3. HDAC2 Expression in Different Groups

**Figure 3:**
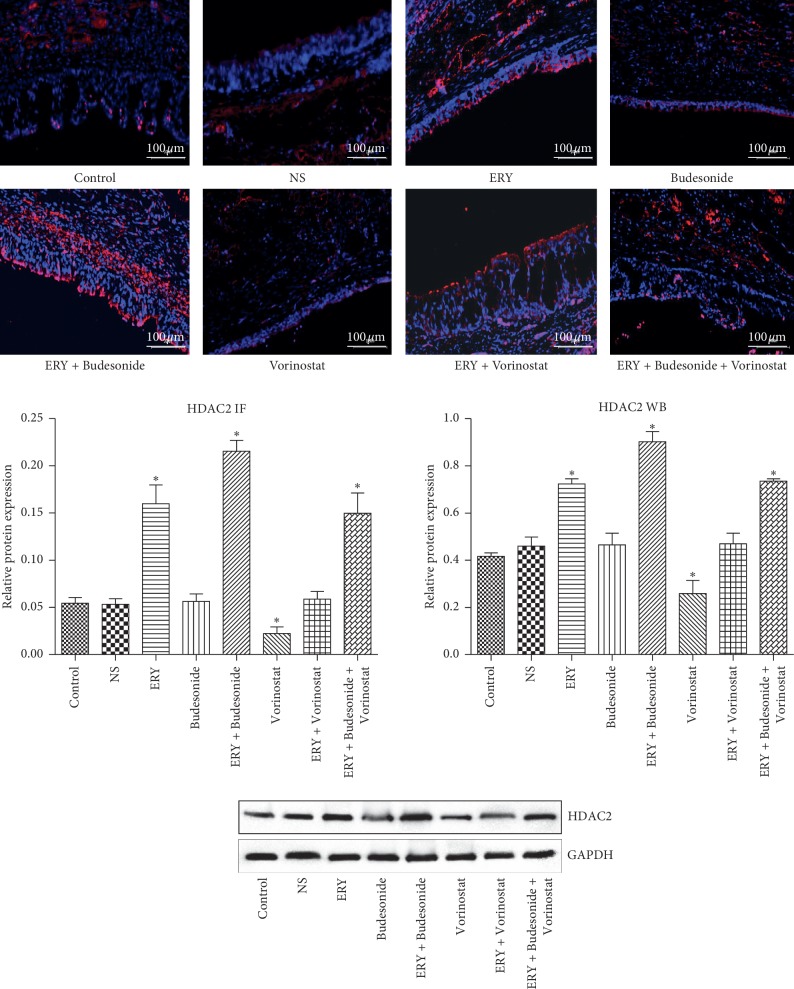
The expression of HDAC2 in the different groups determined by immunofluorescence. Image scale bar, 100 *µ*m. Red fluorescence (Cy3 staining) for the detection of the target protein HDAC2, blue fluorescence (DAPI staining) for the nucleus. Data are presented as the mean ± standard deviation in the corresponding histogram. ^*∗*^*P* < 0.05 vs. the control group. Control: rabbit tracheal stenosis model without treatment; NS: rabbit tracheal stenosis model treated with penicillin; ERY: rabbit tracheal stenosis model treated with erythromycin; Budesonide: rabbit tracheal stenosis model treated with budesonide; Vorinostat: rabbit tracheal stenosis model treated with vorinostat. HDAC2, histone deacetylase-2; IF, immunofluorescence.

As shown in Figure 3, compared with control group, the HDAC2 protein expression increased significantly in ERY group, ERY  + Budesonide group and ERY  +  Budesonide  + Vorinostat group and decreased significantly in Vorinostat group (*P* < 0.05), it was the highest in ERY  +  Budesonide group. There was no difference in HDAC2 protein expression among NS group, Budesonide group and ERY + Vorinostat group (*P* > 0.05).

### 3.4. The Expression of Collagen I and Collagen III in Different Groups

**Figure 4:**
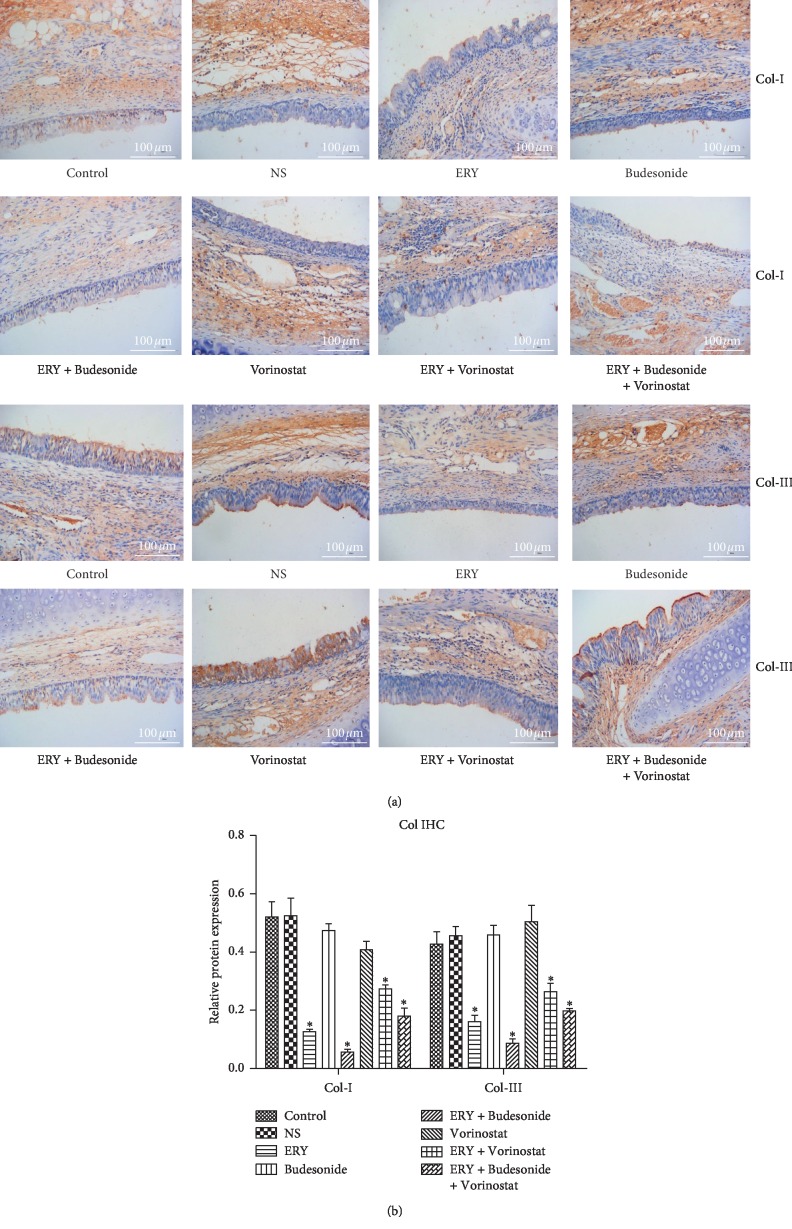
The expression of collagen I and collagen III in the different groups determined by immunohistochemistry. Image scale bar, 100 *µ*m. Brown staining, target protein collagen I and collagen III; blue staining, nucleus. Data are presented as the mean ± standard deviation in the corresponding histogram. ^*∗*^*P* < 0.05 vs. the control group. Control: rabbit tracheal stenosis model without treatment; NS: rabbit tracheal stenosis model treated with penicillin; ERY: rabbit tracheal stenosis model treated with erythromycin; Budesonide: rabbit tracheal stenosis model treated with budesonide; Vorinostat: rabbit tracheal stenosis model treated with vorinostat.

Compared with the control group, the expression of collagen I and III decreased significantly in ERY group, ERY + Budesonide group, ERY + Vorinostat group and ERY + Budesonide + Vorinostat group (Figure 4, *P* < 0.05). There was no difference among NS group, Budesonide group and ERY + Vorinostat group (Figure 4, *P* > 0.05).

### 3.5. The Expression of TGF-*β*1, VEGF and IL-8 in Different Groups

**Figure 5:**
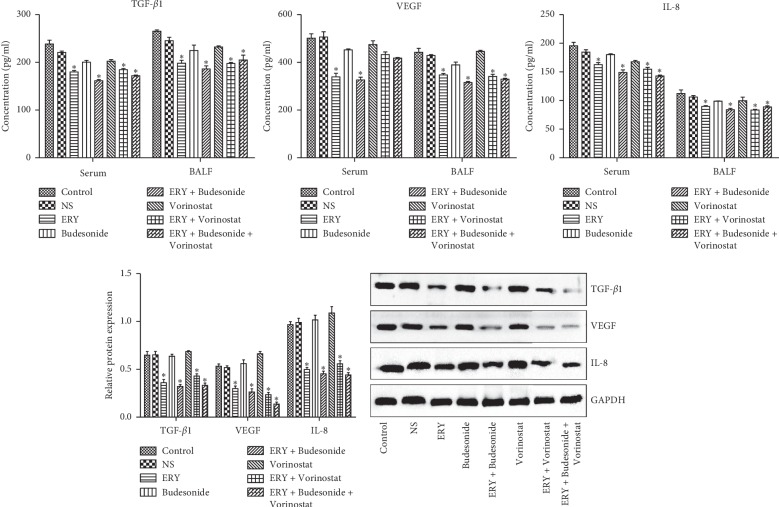
The expression of TGF-*β*1, VEGF and IL-8 in different groups. Control: rabbit tracheal stenosis model without treatment; NS: rabbit tracheal stenosis model treated with penicillin; ERY: rabbit tracheal stenosis model treated with erythromycin; Budesonide: rabbit tracheal stenosis model treated with budesonide; Vorinostat: rabbit tracheal stenosis model treated with vorinostat. ^*∗*^*P* < 0.05 vs. Control group.

Compared with the control group, the expression of TGF-*β*1 and IL-8 in serum decreased significantly in ERY group, ERY + Budesonide group, ERY + Vorinostat group and ERY + Budesonide + Vorinostat group (Figure 5, *P* < 0.05), the expression of VEGF decreased significantly in ERY group and ERY + Budesonide group (Figure 5, *P* < 0.05). The expression of TGF-*β*1, VEGF and IL-8 in BALF decreased significantly in ERY group, ERY + Budesonide group, ERY + Vorinostat group and ERY + Budesonide + Vorinostat group (Figure 5, *P* < 0.05).

## 4. Discussion

Tracheal intubation and tracheotomy are the most common causes of benign tracheal stenosis, their direct injury and possible secondary infection can cause damage to tracheal wall structure, especially to tracheal mucosa. The main treatments for tracheal stenosis including surgical resection of the narrow segment of trachea and end-to-end anastomosis, T-tube placement and endoscopic interventional therapy can re-damage tracheal wall. Moreover, the re-injury is often more serious than the primary injury, which increases the difficulty of follow-up treatment. In the process of injury and repair, various causes cause local and systemic inflammation and induce a variety of inflammatory factors and cytokines [13, 14].

Erythromycin has strong anti-inflammatory activity and immune regulation besides its antimicrobial activity [15]. It can significantly reduce the expression of inflammatory factors such as peroxide and IL-8 by regulating the activity of neutrophils [16]. In recent years, erythromycin's anti-inflammatory activity and immune regulation have achieved good effects in diffuse panbronchiolitis, bronchiectasis, chronic obstructive pulmonary disease, bronchial asthma and other diseases [17–22]. Glucocorticoid is a commonly used anti-inflammatory and immunosuppressive drug. It can bind with glucocorticoid receptors on cell membrane to enter the nucleus, regulate the transcriptional function of many genes, and then regulate cell function [23]. The budesonide used in this study is an anti-inflammatory corticosteroid drug with weak salt corticosteroid activity and strong glucocorticoid activity [24]. Vorinostat capsule is a kind of HDAC2 inhibitor. Erythromycin may increase the activity of HDAC2 and reduce the release of inflammatory factors such as IL-8 by inhibiting the phosphatidylinositol-3 kinase/protein kinase B (PI3K/AKT) pathway, thus enhancing the anti-inflammatory activity of budesonide [25].

The pathological results in this study showed that erythromycin could alleviate the degree of bronchial stenosis and improve the proliferation of mucosal epithelium. Inhaled budesonide alone has no significant improvement on the degree of bronchial stenosis, but combined with erythromycin has more obvious improvement effects.

HDAC2 expression was down-regulated in pulmonary inflammatory diseases such as chronic obstructive pulmonary disease and asthma [26, 27]. This study found that erythromycin and erythromycin combined with budesonide could increase the expression of HDAC2 protein, thereby enhancing the activity of HDAC2. These results suggested that the down-regulation of HDAC2 expression in benign tracheal stenosis animal models may cause local inflammatory reaction disorder and promote the occurrence and development of tracheal stenosis. Low-dose erythromycin has the effect of treating benign tracheal stenosis, which may be related to the up-regulation of HDAC2 expression by low-dose erythromycin, and then inhibit the inflammatory disorder during tracheal injury repair.

It was reported that erythromycin inhibited the production of Col-I and Col-III by human nasal polyp fibroblasts through antioxidant effect [28]. This study found that erythromycin alone and erythromycin combined with other drugs could reduce the expression of Col-I and Col-III, it could effectively reduce tracheal stenosis after tracheal injury. TGF-*β* is a cytokine superfamily, in which TGF-*β*1 plays a major role. TGF-*β*1 is a growth factor closely related to scar formation and widely participates in various processes of wound healing. Fibroblasts are the main target of TGF-*β*1, which can promote the synthesis of extracellular matrix and inhibit its decomposition, so they are one of the most important factors to promote fibrosis [29, 30]. The expression of TGF-*β*1 increased in tracheal scar tissue of patients with benign tracheal stenosis, which also confirmed that TGF-*β*1 was closely related to the scar formation of benign tracheal stenosis [31]. The up-regulation of TGF-*β*1 expression promoted angiogenesis and fibroblast proliferation through the TGF-*β*1/Smads signaling pathway, and promoted the synthesis of extracellular matrix, especially collagen [32, 33].

The VEGF family mainly includes VEGF-A, VEGF-B, VEGF-C, VEGF-D and placental growth factor (PGF). VEGF-A is the earliest and most important growth factor for promoting angiogenesis [34]. The receptor phosphorylates itself after binding with the VEGF family, activates PI3K/AKT and Ras/MAPK signaling pathways, promotes endothelial cell differentiation, migration and proliferation, and thus produces a large number of new blood vessels to promote wound healing. VEGF can be expressed in fibroblasts, keratinocytes, vascular endothelial cells and macrophages, its main biological function is to promote angiogenesis and regulate fibroblast activity [35]. IL-8 is a multifunctional inflammatory factor, which is widely involved in acute and chronic inflammation. It can promote fibroblast proliferation, promote collagen synthesis, inhibit collagen fiber decomposition, promote the deposition of extracellular matrix, and then facilitate the formation of granulation tissue and fibrosis [36]. Study showed that the expression of IL-8 gene in fibroblasts of scar tissue was significantly up-regulated than that in normal tissue [37]. In this study, the concentration of cytokines such as TGF-*β*1, VEGF, IL-8 and inflammatory factors in each experimental group treated by erythromycin was significantly lower than that in other non-erythromycin groups, which also showed that erythromycin had anti-inflammatory effect.

## 5. Conclusions

In a word, erythromycin may inhibit inflammation after tracheobronchial mucosal injury by up-regulating the expression of HDAC2, down-regulating the expression of inflammatory factors, inhibiting the excessive proliferation and fibrosis of granulation tissue, promotes wound healing and alleviates tracheobronchial stenosis. When erythromycin combined with budesonide and other glucocorticoids and antibiotics, it had a good synergistic effect. However, vorinostat attenuated this effect of erythromycin by down-regulating the expression of HDAC2. Erythromycin may have good clinical application prospects in preventing tracheal stenosis and restenosis after tracheal injury.

## Data Availability

The data used to support the findings of this study are available from the corresponding author upon request.

## References

[1] Shitrit D., Kuchuk M., Zismanov V., Rahman N. A., Amital A., Kramer M. R. (2010). Bronchoscopic balloon dilatation of tracheobronchial stenosis: long-term follow-up. *European Journal of Cardio-Thoracic Surgery*.

[2] Liang Y.-L., Liu G.-N., Zheng H.-W. (2015). Management of benign tracheal stenosis by small-diameter tube-assisted bronchoscopic balloon dilatation. *Chinese Medical Journal*.

[3] Smith M. E., Elstad M. (2009). Mitomycin C and the endoscopic treatment of laryngotracheal stenosis: are two applications better than one?. *The Laryngoscope*.

[4] Ferreira S., Nogueira C., Oliveira A., Neves S., Almeida J., Moura e Sá J. (2010). Técnicas de dilatação broncoscópica e aplicação tópica de mitomicina C no tratamento da estenose traqueal pós-entubação-a propósito de dois casos clínicos. *Revista Portuguesa de Pneumologia*.

[5] Simpson C. B., James J. C. (2006). The efficacy of mitomycin-C in the treatment of laryngotracheal stenosis. *The Laryngoscope*.

[6] Zhu G. H., Ng A. H. C., Venkatraman S. S. (2011). A novel bioabsorbable drug-eluting tracheal stent. *The Laryngoscope*.

[7] Shin J. H., Song H.-Y., Seo T.-S. (2005). Influence of a dexamethasone-eluting covered stent on tissue reaction: an experimental study in a canine bronchial model. *European Radiology*.

[8] Yao X. P., Li Q., Bai C. (2005). Restenosis and its management after metallic stents implantation in benign tracheal and main bronchial stenosis. *Zhonghua Nei Ke Za Zhi*.

[9] Yan H., Yi S., Zhuang H., Wu L., Wang D. W., Jiang J. (2017). Sphingosine-1-phosphate ameliorates the cardiac hypertrophic response through inhibiting the activity of histone deacetylase-2. *International Journal of Molecular Medicine*.

[10] Sun X.-J., Li Z.-H., Zhang Y. (2015). Combination of erythromycin and dexamethasone improves corticosteroid sensitivity induced by CSE through inhibiting PI3K-*δ*/Akt pathway and increasing GR expression. *American Journal of Physiology-Lung Cellular and Molecular Physiology*.

[11] Enyuan Q., Mingpeng X., Luoman G. (2018). Erythromycin combined with corticosteroid reduced inflammation and modified trauma-induced tracheal stenosis in a rabbit model. *Therapeutic Advances in Respiratory Disease*.

[12] Xie M., Kong Y., Tan W. (2014). Histone deacetylase inhibition blunts ischemia/reperfusion injury by inducing cardiomyocyte autophagy. *Circulation*.

[13] Puyo C. A., Dahms T. E. (2012). Innate immunity mediating inflammation secondary to endotracheal intubation. *Archives of Otolaryngology-Head & Neck Surgery*.

[14] Zhang J., Li Q., Bai C., Han Y., Huang Y. (2009). Inhalation of TGF-*β*1 antibody: a new method to inhibit the airway stenosis induced by the endobronchial tuberculosis. *Medical Hypotheses*.

[15] Giamarellos-Bourboulis E. J. (2008). Macrolides beyond the conventional antimicrobials: a class of potent immunomodulators. *International Journal of Antimicrobial Agents*.

[16] Lin H.-C., Wang C.-H., Liu C.-Y., Yu C.-T., Kuo H.-P. (2000). Erythromycin inhibits *β*2-integrins (CD11b/CD18) expression, interleukin-8 release and intracellular oxidative metabolism in neutrophils. *Respiratory Medicine*.

[17] Serisier D. J., Martin M. L., McGuckin M. A. (2013). Effect of long-term, low-dose erythromycin on pulmonary exacerbations among patients with non-cystic fibrosis bronchiectasis. *JAMA*.

[18] Tsang K. W. T., Ho P.-I., Chan K.-N. (1999). A pilot study of low-dose erythromycin in bronchiectasis. *European Respiratory Journal*.

[19] Chuang M.-C., Chou Y.-T., Lin Y.-C., Hsieh M.-J., Tsai Y.-H. (2016). Diffuse panbronchiolitis-the response and recurrence after erythromycin therapy. *Journal of the Formosan Medical Association*.

[20] Park S.-J., Lee Y.-C., Rhee Y.-K., Lee H.-B. (2004). The effect of long-term treatment with erythromycin on Th1 and Th2 cytokines in diffuse panbronchiolitis. *Biochemical and Biophysical Research Communications*.

[21] Korematsu S., Yamamoto K., Nagakura T. (2010). The indication and effectiveness of low-dose erythromycin therapy in pediatric patients with bronchial asthma. *Pediatric Allergy and Immunology*.

[22] Miyatake H., Taki F., Taniguchi H., Suzuki R., Takagi K., Satake T. (1991). Erythromycin reduces the severity of bronchial hyperresponsiveness in asthma. *Chest*.

[23] Zhou F., Medh R. D., Thompson E. B. (2000). Glucocorticoid mediated transcriptional repression of c-myc in apoptotic human leukemic CEM cells. *The Journal of Steroid Biochemistry and Molecular Biology*.

[24] Thomson A., Sadowski D., Jenkins R., Wild G. (1997). Budesonide in the management of patients with crohn’s disease. *Canadian Journal of Gastroenterology*.

[25] Yao K. L., Liu G. N., Huang S. M., Liu T., Li Y. (2016). Relationship between expression of HDAC2, IL-8, TNF-alpha in lung adenocarcinoma tissues and smoking. *Zhonghua Yi Xue Za Zhi*.

[26] Adenuga D., Yao H., March T. H., Seagrave J., Rahman I. (2009). Histone deacetylase 2 is phosphorylated, ubiquitinated, and degraded by cigarette smoke. *American Journal of Respiratory Cell and Molecular Biology*.

[27] Bhavsar P., Ahmad T., Adcock I. M. (2008). The role of histone deacetylases in asthma and allergic diseases. *Journal of Allergy and Clinical Immunology*.

[28] Ortega M. A., Asúnsolo Á, Álvarez-Rocha M. J. (2018). Remodelling of collagen fibres in the placentas of women with venous insufficiency during pregnancy. *Histology and Histopathology*.

[29] Sato M. (2006). Upregulation of the wnt/*β*-catenin pathway induced by transforming growth factor-*β* in hypertrophic scars and keloids. *Acta Dermato-Venereologica*.

[30] Jagadeesan J., Bayat A. (2007). Transforming growth factor beta (TGF*β*) and keloid disease. *International Journal of Surgery*.

[31] Lee Y.-C., Hung M.-H., Liu L.-Y. (2011). The roles of transforming growth factor-*β*_1_ and vascular endothelial growth factor in the tracheal granulation formation. *Pulmonary Pharmacology & Therapeutics*.

[32] Kryger Z. B., Sisco M., Roy N. K., Lu L., Rosenberg D., Mustoe T. A. (2007). Temporal expression of the transforming growth factor-beta pathway in the rabbit ear model of wound healing and scarring. *Journal of the American College of Surgeons*.

[33] Yu H., Bock O., Bayat A., Ferguson M. W. J., Mrowietz U. (2006). Decreased expression of inhibitory SMAD6 and SMAD7 in keloid scarring. *Journal of Plastic, Reconstructive & Aesthetic Surgery*.

[34] Arjaans M., Schröder C. P., Oosting S. F., Dafni U., Kleibeuker J. E., de Vries E. G. E. (2016). VEGF pathway targeting agents, vessel normalization and tumor drug uptake: from bench to bedside. *Oncotarget*.

[35] Yun S. P., Lee M. Y., Ryu J. M., Song C. H., Han H. J. (2009). Role of HIF-1*α* and VEGF in human mesenchymal stem cell proliferation by 17*β*-estradiol: involvement of PKC, PI3K/Akt, and MAPKs. *American Journal of Physiology-Cell Physiology*.

[36] David J. M., Dominguez C., Hamilton D. H., Palena C. (2016). The IL-8/IL-8R axis: a double agent in tumor immune resistance. *Vaccines*.

[37] Shoshan E., Braeuer R. R., Kamiya T. (2016). NFAT1 directly regulates IL8 and MMP3 to promote melanoma tumor growth and metastasis. *Cancer Research*.

